# A comparative study of the gut microbiome in Egyptian patients with Type I and Type II diabetes

**DOI:** 10.1371/journal.pone.0238764

**Published:** 2020-09-09

**Authors:** Sahar Radwan, Darby Gilfillan, Bridget Eklund, Hend M. Radwan, Nagwan G. El Menofy, Justin Lee, Marylee Kapuscinski, Zaid Abdo

**Affiliations:** 1 Microbiology and Immunology Department, Faculty of Pharmacy (Girls), Al-Azhar University, Cairo, Egypt; 2 Department of Microbiology Immunology and Pathology, Colorado State University, Fort Collins, Colorado, United States of America; Cairo University, EGYPT

## Abstract

**Introduction:**

Diabetes remains a growing public health concern in Egypt, as prevalence of Type II diabetes (TIID) has nearly tripled there in the last two decades. Egypt was ranked ninth worldwide in number of diabetes cases, with prevalence of 15.56% among adults. Recent studies have proposed that disturbance of gut microbiota could influence TIID development and indicated associations between a reduced diversity in microbiomes and Type I diabetes (TID). In the present study, we investigated the composition and abundance of the bacterial microbiome in disease state (TID and TIID) of Egyptian patients. Our goal in this study was to characterize features of the gut microbiota and possible differences associated with TID and TIID in this population.

**Methods:**

DNA was extracted from fecal samples taken from 22 TID and 18 TIID outpatients of Al-Hussein hospital, Cairo, Egypt. 16S rRNA amplicon sequencing was used to characterize the bacterial taxa and these reads were processed using the software mothur with analysis utilizing packages vegan, phyloseq and metagenomSeq in R.

**Results and conclusions:**

Our results highlighted a significant increase in abundance of Gram negative, potentially opportunistic pathogenic taxa (*Pseudomonas*, *Prevotella*) in all diabetic groups, compared to the control. Lipopolysccharide (LPS), a component of the gram-negative bacterial wall, can activate local immune response and may result in low-grade systemic inflammation contributing to insulin resistance. The gram-positive *Gemella*, which is associated with increased risk to diabetes, also had a significant increase in abundance in all diabetic groups, compared to the control. In contrast, the commensal bacterial taxa *Turicibacter*, *Terrisporobacter* and *Clostridium* were found to be more abundant in the control group than in TID. Further studies are needed to understand the role of these taxa in health and disease. Lower Richness and low Shannon diversity, though not statistically significant, were observed for TID subjects with no glucose control and with onset of liver disease or hypertension compared to other subjects. In addition, large variation in alpha diversity within the control group could also be observed. Future studies will include larger samples sizes to further elucidate these findings, as well as possible metagenomic studies to examine the intriguing function of significant microbes.

## Introduction

Diabetes mellitus (DM) is a diverse metabolic disorder characterized by elevated blood sugar levels as a result of deficiency of insulin secretion, defective insulin action or both [1]. DM can cause complications if uncontrolled, including, stroke, cardiovascular disease and kidney failure [2]. Globally, DM is the ninth major cause of death; one in eleven adults worldwide have DM [3].The International Diabetes Federation (IDF) ranked Egypt ninth worldwide in number of diabetes cases, with prevalence of 15.56% among adults [4,5].

Diabetes cases can be categorized into 3 classes [6]. Type I diabetes (TID) is characterized by the autoimmune destruction of pancreatic B cells. Over the past 50 years there was an increase in incidence of TID that may be attributed to genetic predisposition and several environmental factors including stress and viral infections [7,8]. Type II diabetes (TIID) represents about 90% of all diabetes cases worldwide. It is associated with an unhealthy lifestyle and diet, obesity, lack of exercise and physical activity, in addition to other poor dietary habits [9]. The third class is known as MODY (Maturity onset diabetes of the young). is a rare but increasingly recognized cause of diabetes in young people. MODY is commonly misdiagnosed as type 1 or type 2 diabetes and, as a result, patients are often inappropriately managed with insulin when they can be more effectively managed with oral sulfonylureas [6].

Recent research, driven by advances in high throughput 16S rRNA amplicon sequencing and shotgun metagenomics, has established that the gut microbiome includes100-fold or more genes than the human genome [10–12]. These microbial genes are considered key to metabolic processes with impact to the host, including catabolism of dietary fibers to short-chain fatty acids, amino acid and vitamin biosynthesis, as well as aiding the production of neurotransmitters and hormones [10]. The previous decade witnessed many studies that aimed to explain the role of the gut microbiota in TIID and glycemic control. Recent studies have proposed that disturbance of gut microbiota could influence TIID development [13,14]. Significance of diversity of the microbiota in controlling metabolic processes was revealed by Le Chatelier et al. [15] and Cotillard et al. [16] who reported association between low diversity of the gut microbiome with obesity, non-alcoholic fatty liver disease and a higher prevalence of insulin resistance [15,16].

Type I diabetes is associated with a well-known genetic mutation in the human leukocyte antigen genes. High incidence of this disease can also be attributed to environmental factors [17]. A study of eight children, four with newly developed TID and four matched controls, found differences in the composition of the gut metagenome between the two groups and reduced diversity in the TID-associated microbiomes [18]. Another study in non-obese diabetic (NOD) mice have demonstrated that germ-free NOD mice are more likely to have diabetes, suggesting a role for the gut microbiota in the development of autoimmune diabetes [19].

To better understand the features of gut microbiota in TID and TIID, here we investigated the gut microbiome of 47 Egyptian citizens (7 healthy controls, 22 TID and 18 TIID), using the conserved V4 region of the bacterial 16S ribosomal DNA. We compared the composition, diversity and richness (number of species) of the fecal microbial ecosystem of healthy, TID and TIID patients. To our knowledge, this is the first metagenomic study comparing the gut microbiome among TID and TIID patients in Egypt.

## Materials and methods

### Ethical considerations

Study protocol was approved by the Ethics Committee of the Faculty of Pharmacy at Al-Azhar University in Cairo, Egypt. All participants provided written informed consent prior to sample collection.

### Human subjects

This study included 47 subjects (7 healthy controls, 22 TID and 18 TIID). TID and TIID subjects were further stratified by whether they were treated or not (Controlled versus Uncontrolled diabetes symptoms) and by secondary disease onset, if present (S1 Table). All study groups were matched for age, sex and type of medication. Subjects were excluded if they received antibiotics within one month prior to sampling.

### Sample collection and DNA extraction

Sample collection was performed on site in Egypt as described previously [14]. Briefly, fecal samples were collected immediately after defecation and placed in an Eppendorf tube pre-prepared with saline solution. These samples were preserved at –20˚C until processed. DNA was extracted from approximately 0.3–0.5g from each stool sample using QIAamp DNA Stool Mini Kit (Qiagen, Hilden, Germany) according to the manufacturer’s instructions. DNA concentration and quality were evaluated utilizing a Qubit 2.0 fluorometer (Invitrogen,California USA) and gel electrophoresis.

### DNA sequencing

Isolated microbial DNA from each sample was used for amplification of the hypervariable region 4 (V4) of the 16S rRNA gene, as described previously [20]. Briefly, the universal 515F/806R primer set was used in a single-step 30-cycle PCR reaction to generate multiplexeddual-indexed library molecules. Controls included the ZymoBIOMICS^TM^ D6311 Microbial DNA Community Standard II (mock community) and a no-template negative control, which was included in each PCR plate. An additional PCR reaction was conducted using P5 and P7 library amplification primers with 15 cycles to help further amplify these library molecules. To confirm amplification, libraries were visualized on an agarose gel. Library molecules were purified using Sera-Mag beads (GE Life Sciences) to select for DNA fragments larger than 150bp. Purified library molecules were quantified using Quant-iT Broad-Range dsDNA kit (Invitrogen) according to manufacturer’s protocols. An equimolar concentration of each library was combined, and the final pool was brought to a concentration of 4 nM.

The 4 nM pooled library was quality controlled with the NEBNext Quant Kit for Illumina E7630 (New England Biolabs) according to manufacturer’s protocols. The library was diluted and denatured according to Illumina’s MiSeq System Denature and Dilute Libraries Guide number 15039740–10. Briefly, the library was mixed with freshly prepared 0.2 N NaOH for denaturation and diluted to a final concentration of 8pM. A PhiX control was denatured and diluted to the same loading concentration as the amplicon library to add necessary diversity to the library. The final library mixture contained 10% PhiX. The library was sequenced on an Illumina MiSeq at Colorado State University’s Next Generation Sequencing Core Facility using a MiSeq Reagent Kit v2 500-cycle, resulting in paired-end 2x250 base-pair reads. The resulting raw sequence data are publicly available on the National Center for Biotechnology Information’s (NCBI) Sequence Read Archive (SRA) repository, accession number PRJNA629382 and the associated sample key is also provided in S2 Table.

### Data processing and bioinformatics

Demultiplexing and base calling were both performed using bcl2fastq Conversion Software v2.18 (Illumina, Inc.). We utilized the software FastQC [21] for quality assessment and the software trimmomatic (version 0.39) [22] with sliding window of size 4 and cutoff quality at PHRED 30, to filter the resulting sequence data selecting for high quality reads for further analyses. A minimum length of 150 base-pairs was also used to guarantee an overlap between the forward and reverse reads required for constructing the contigs used in further processing. Further data processing was conducted using mothur [23] version 1.40.5 and utilizing an adjustment to the developers’ standard operating procedure (SOP) for OTU clustering and classification [20]. Adjustments included the use of UCHIME for de novo chimera detection and the use of USEARCH, utilizing the dgc (distance-based greedy clustering) option, for Clustering (adjusted SOP can be found at https://github.com/Abdo-Lab/Microbiome-Analysis-Scripts/blob/master/PE-de-novo-processing.pl). Groups that were at least 97% similar were classified to belong to the same operational taxonomic unit (OTU). We utilized the SILVA (nr_v132) database [24] for bacterial taxonomic classification. Rarefaction curves were generated using the package 'vegan' [25] as implemented in R version 3.6.1 to assess diversity and suitability of depth of coverage per sample. The resulting OTU table and taxonomic assignment were utilized in further data analyses.

### Statistical analysis

Alpha diversity measures were calculated and included rarefied richness, as computed using the package 'vegan' in R [25], (we refer to it as Richness here after).The Shannon diversity index was computed utilizing the R package phyloseq [26]. We used an analysis of variance (ANOVA) to assess significance of the treatment levels.

Nonmetric Multidimensional Scaling (NMDS) was used on the OTU level to assess possible trends and clustering in the microbial community structure per treatment condition (Beta diversity). NMDS was performed using the vegan package [25] and utilizing Bray-Curtis dissimilarity and was based on data normalized utilizing cumulative sum scaling (CSS) [27]. We plotted the 95% confidence ellipsoids utilizing the standard error within each treatment group. Distance based; permutation based multivariate analysis of variance (PerMANOVA) [28] was employed to assess significant differences between the microbial communities per treatment level.

Utilizing relative abundance data based on the resulting OTU table, bar graphs were generated using the ggplot2 package [29] in R for taxa that were observed with relative abundance > 1% at the family level to describe the microbial community structure per time point under each of the treatments. The package metagenomeSeq [27] was used to identify the OTUs driving differences between the treatment levels. We fit a cell means model accounting for possible differences between treatment means. This model fits a mean per each treatment and compares against a null model that assumes no differences between all means. OTUs chosen part of this analysis were selected to be present in at least 10 samples with at least one observed sequence read per sample. Log-fold-changes were compared between treatments using empirical Bayes’ moderated t values calculated utilizing the function eBayes in the package limma [30] and using false-discovery-rate (FDR) [31] adjusted p-values utilizing the R package fdrftool [32]. Log-fold-change differences with an adjusted p-values less than 0.05 were deemed significant.

## Results

In this study a total of 47 human fecal samples were analyzed by 16S rRNA amplicon sequencing targeting the V4 region to investigate the microbiota composition between healthy, TID and TIID subjects.

### Human subjects

Patients were categorized into groups according to type of diabetes, controlled blood glucose level, age and other medical condition. S1 Table summarizes different factors associated with the human subjects.

### Preprocessing of DNA sequence data

DNA from fifty-three samples was sequenced including the aforementioned 47 samples obtained from the subjects associated with this study along with four negative no template controls (NTC) and two positive ZymoBIOMICS^TM^ D6311 standard controls utilizing a log distribution of the taxa resulting in a ladder of known taxa observed at different decreasing abundances. We observed a range between 6 and 219,746 reads per sample after pre-processing using trimmomatic and mothur as described above and a total of 3,405 operational taxonomic units (OTUs) in all samples. Seven of the 47 samples resulted in fewer reads than the negative controls, ranging between 6 and 798 reads, and hence were dropped from further analysis. These samples did not have sufficient DNA for sequencing to start with. We chose a cutoff of 15 reads that was subtracted from each OTU count per sample to further account for possible contamination in the negative controls. This cutoff resulted in a reduction of observed putative contaminants in the negative controls from 38 to 4, two of which were observed in only one of these samples. This cutoff resulted in observing only five of the eight bacterial taxa present in the positive control missing those taxa with abundance equal to or less than 0.01% of the total. The resulting OTU table after this rigorous filtering included 619 OTUs with total number of reads per sample ranging from 6,219 to 214,764 belonging to 40 samples as described in Table 1. S1 Fig shows the rarefaction curves associated with these samples indicating that the depth of sequencing for these samples was adequate to capture the underlying diversity.

**Table 1:** Summary of the samples surviving further processing.

Item	TID No. %)	TIID No. (%)	Healthy control No. (%)
**Controlled blood glucose level- Diabetic only**	7	6	
**Not controlled blood glucose level- Diabetic with liver diseases**	4	1	
**Not controlled blood glucose level- Diabetic with hypertension**	3	2	
**Not controlled blood glucose level- Diabetic only**	4	6	
**Total**	18	15	7

TID; Type I diabetes, TIID; Type II diabetes

### Statistical analysis

Given the small sample size we combined the data across gender and age. We also combined the not-controlled blood level samples with hypertension and liver disease into one category that we referred to as not-controlled diseased. This resulted in seven treatment levels that we compared and presented in Table 2.

**Table 2:** Abbreviation of different study groups.

Abbreviation	Treatment Description
**TIC**	*Type I diabetic with controlled blood glucose*
**TIIC**	*Type II diabetic with controlled blood glucose*
**TIND**	*Type I diabetic with no blood glucose control and disease*
**TIIND**	*Type II diabetic with no blood glucose control and disease*
**TINN**	*Type I diabetic with no blood glucose control and no disease*
**TIINN**	*Type II diabetic with no blood glucose control and no disease*
**C**	*Healthy controls*

#### Alpha diversity and beta diversity

**Fig 1:**
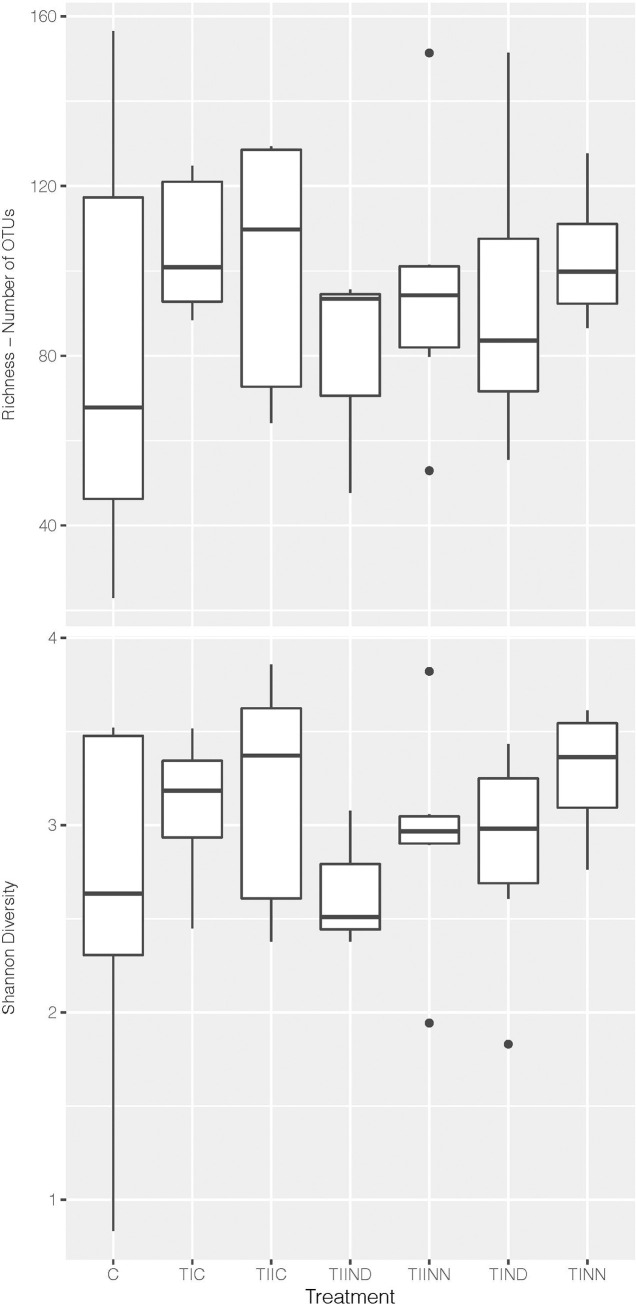
Richness and Shannon diversity per treatment level indicating qualitatively lower diversity for treatment TIIND and highlighting the high variability in the control group (C). * Abbreviations in Table 2.

A qualitatively lower Richness and low Shannon diversity was observed for subjects with no glucose control and with onset of liver disease or hypertension (TIIND) compared to other subjects (Fig 1). Large variation in alpha diversity within the control group can also be observed from Fig 1. An analysis of variance applied to both Richness and Shannon diversity showed no significance and could not identify possible significant differences between the treatments (F = 0.52, p-value = 0.79 and F = 0.76, p-value = 0.60 for Richness and Shannon diversity, respectively).

Trends in Beta diversity also indicated no significant differences between the treatments reflected in overlap in the confidence ellipsoids in the nonmetric multidimensional scaling (NMDS) plot for all treatments (S2 Fig). This lack of a significance was also highlighted by an insignificant outcome (p-value = 0.86) of a perMANOVA test aimed to assess differences between the different treatment levels described above. Comparing the type I, type II and the control groups did not result in any significant difference (perMANOVA p-value = 0.34).

**Fig 2:**
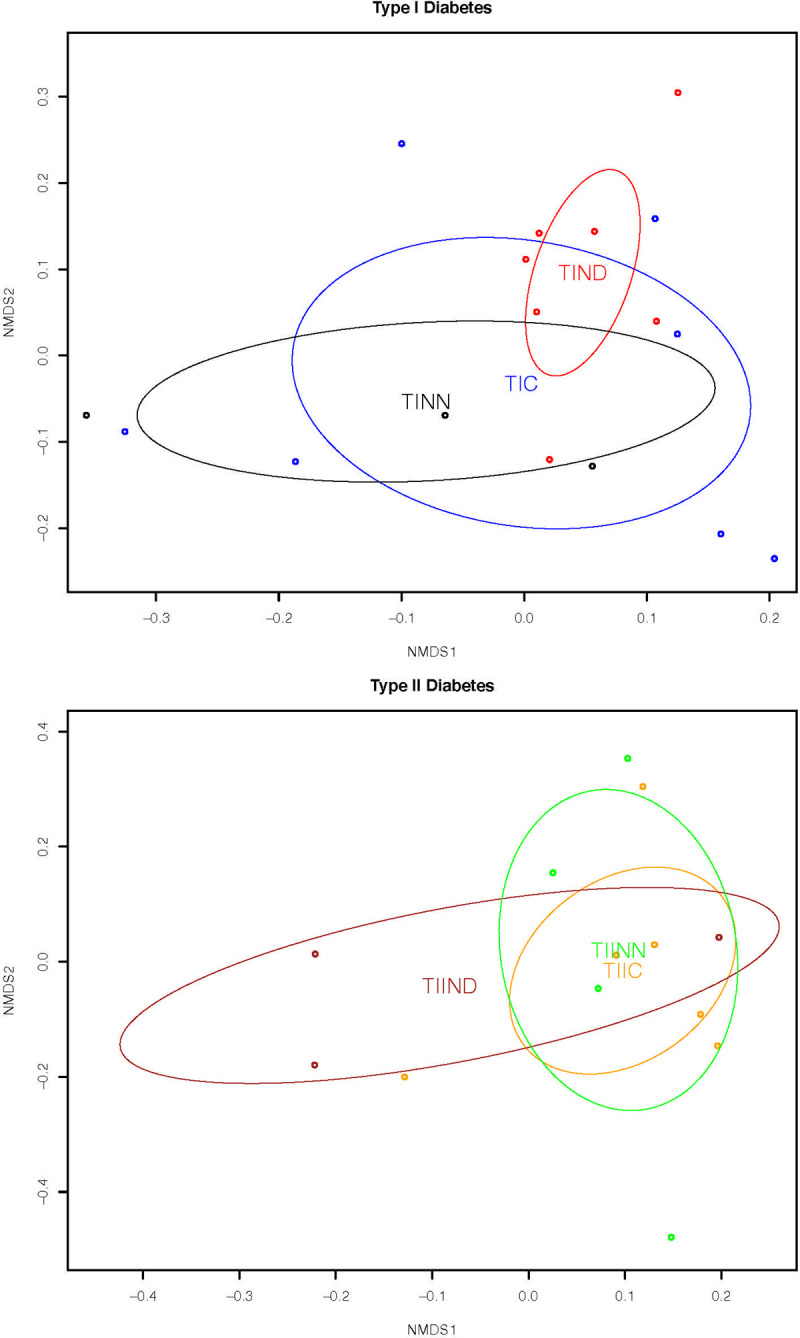
NMDS plot comparing treatments within the TID (A) and within TIID (B) diabetes patients indicating possible qualitative separation between TIND and TINN treatments within the TID subjects. Data points are marked with “*”. * Abbreviations in Table 2.

The NMDS plot in Fig 2 compares treatments within TID and TIID patients. The figure indicates a qualitative difference, although not significant, in beta diversity between TID patients where glucose levels were not controlled with and without onset of disease. No trends were observed for patients of TIID and no differences were observed between patients of TID and TIID per treatment.

#### Comparing treatment effects by OUT

Nineteen families were identified with abundance more than 1% per sample within each of the treatment levels. S3 Fig shows these families aggregated over samples within each treatment level. These families mainly belonged to phylum Firmicutes (Ruminococcaceae, Veillonellaceae, Streptococcaceae, Peptostreptococcaceae, Listeraceae, Leuconostocaceae, Lactobacillaceae, Lachnospiraceae, Eryslipelotrichaceae, Clostridiaceae, Christensenellaceae, Bifidobacteriaceae, Akkermanalaceae and the Gram negative Acidaminococcaceae).

**Fig 3:**
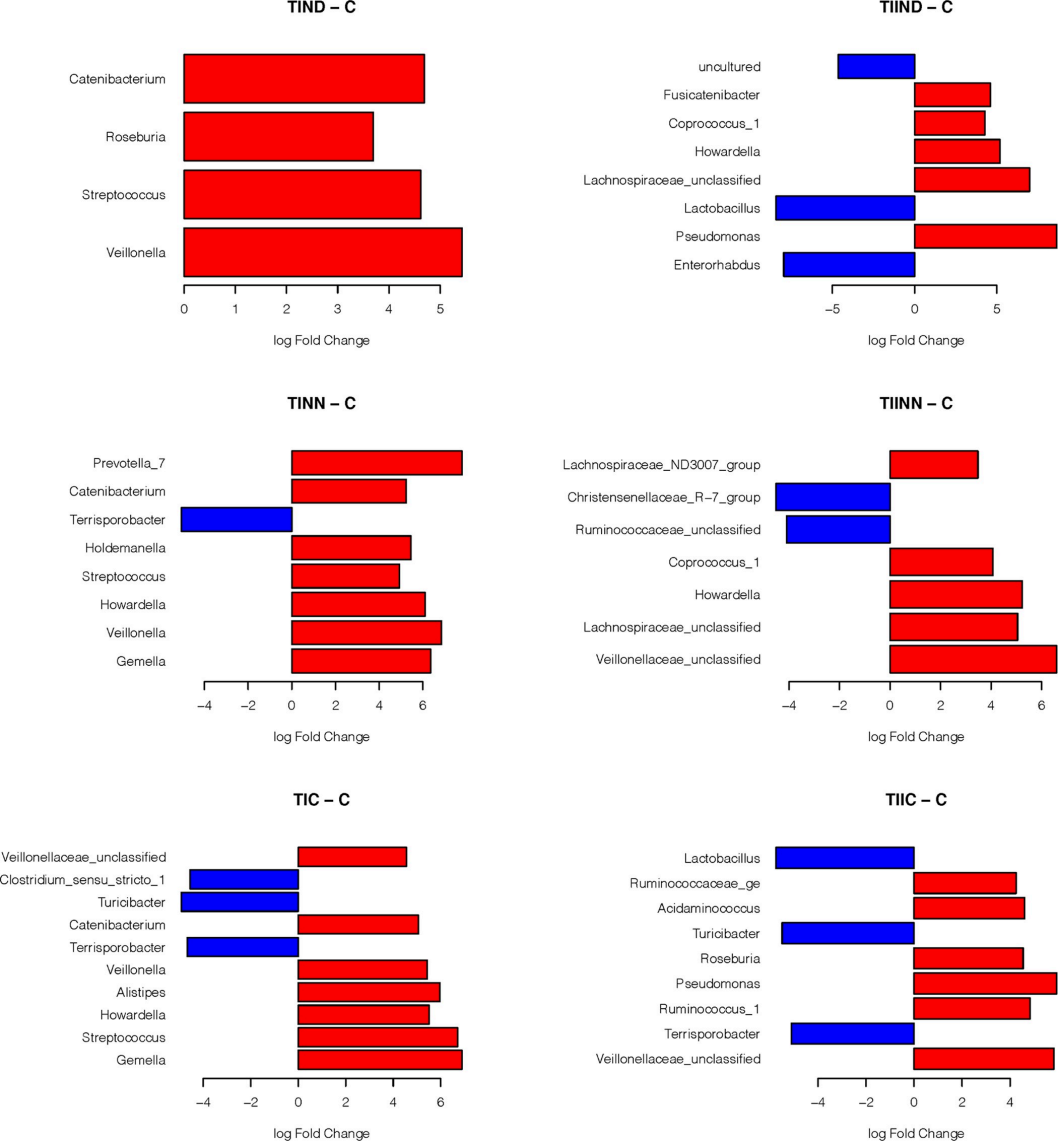
Log-fold-change of OTUs as compared between the different treatments and the control group. Detailed results of the statistical testing are in S3 Table. * Abbreviations in Table 2.

Only one hundred and forty-five OTUs were present in 10 samples with number of reads greater than 1 meeting the rule we set to limit sparsity of the data used in metagenomeSeq analyses. Forty-three of these OTUs where identified as being significantly different at the 0.05 level in at least one of fifteen pair wise comparisons (range from 4 to 9 per comparison after correcting for multiple testing using FDR as described above). Comparisons included testing the log-fold-changes between all treatments and the control, comparing treatments withinTID subjects, comparing treatments within TIID subjects, and comparing matched treatments between TID and TIID subjects (for example, TIND against TIIND). S3 Table provides a summary of these comparisons. Fig 3 shows the significantly different log-fold-change of OTUs as compared between the different treatments and the control group. The red bars represent an increased abundance in the treatment and the blue bars represent increased abundance in the control. We note that *Veillonella*, *Streptococcus*, *Catenibacterium* and *Roseboria* showed significantly higher log fold change in TIND than in the control group. While in TIIND, *Pseudomonas*, Lachnospiraceae *and Howardella* were more significant compared to the control group that showed significant increase in *Lactobacillus*. For TINN, *Prevotella and Veillonella* were significantly higher than the control group which showed higher abundance of *Terrisporobacter*. TIINN presented higher abundance for Veillonellaceae, Lachnospiraceae and Howardella, while in the control group, Ruminococcaceae and Christensenellaceae showed significantly higher abundance than the TIINN. Comparing control group with both types of diabetes that had controlled blood glucose level were nearly the same as *Terrisporobacter* and *Turicibacter* were the most abundant among the control group, while *Gemella*, *Streptococcus* and *Alistipes* were more abundant in TIC. On the other hand, TIIC showed higher abundance of *Pseudomonas and* Veillonellaceae.

**Fig 4:**
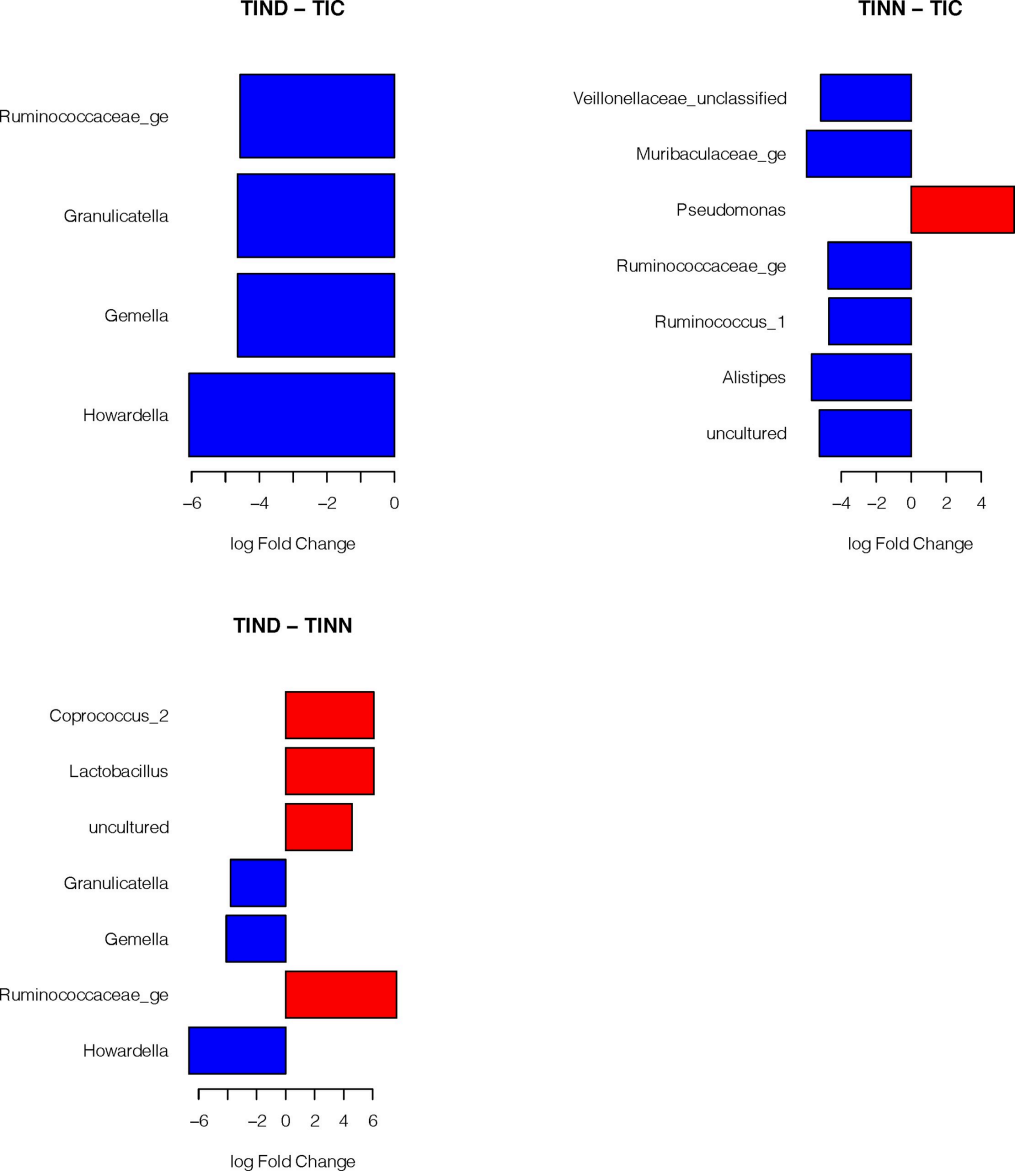
Log-fold-change between the different treatments for the type I diabetic subjects. Detailed results of the statistical testing are in S3 Table. * Abbreviations in Table 2.

The different treatments for the TID subjects are presented in Fig 4. We note that the control group always shows more variability than TID of different treatments and higher abundance in beneficial bacterial families such as Ruminococcaceae and Veillonellaceae. Also, it was observed that *Pseudomonas* showed higher abundance in TINN than TIC. *Pseudomonas* is well documented as opportunistic pathogenic bacteria.

**Fig 5:**
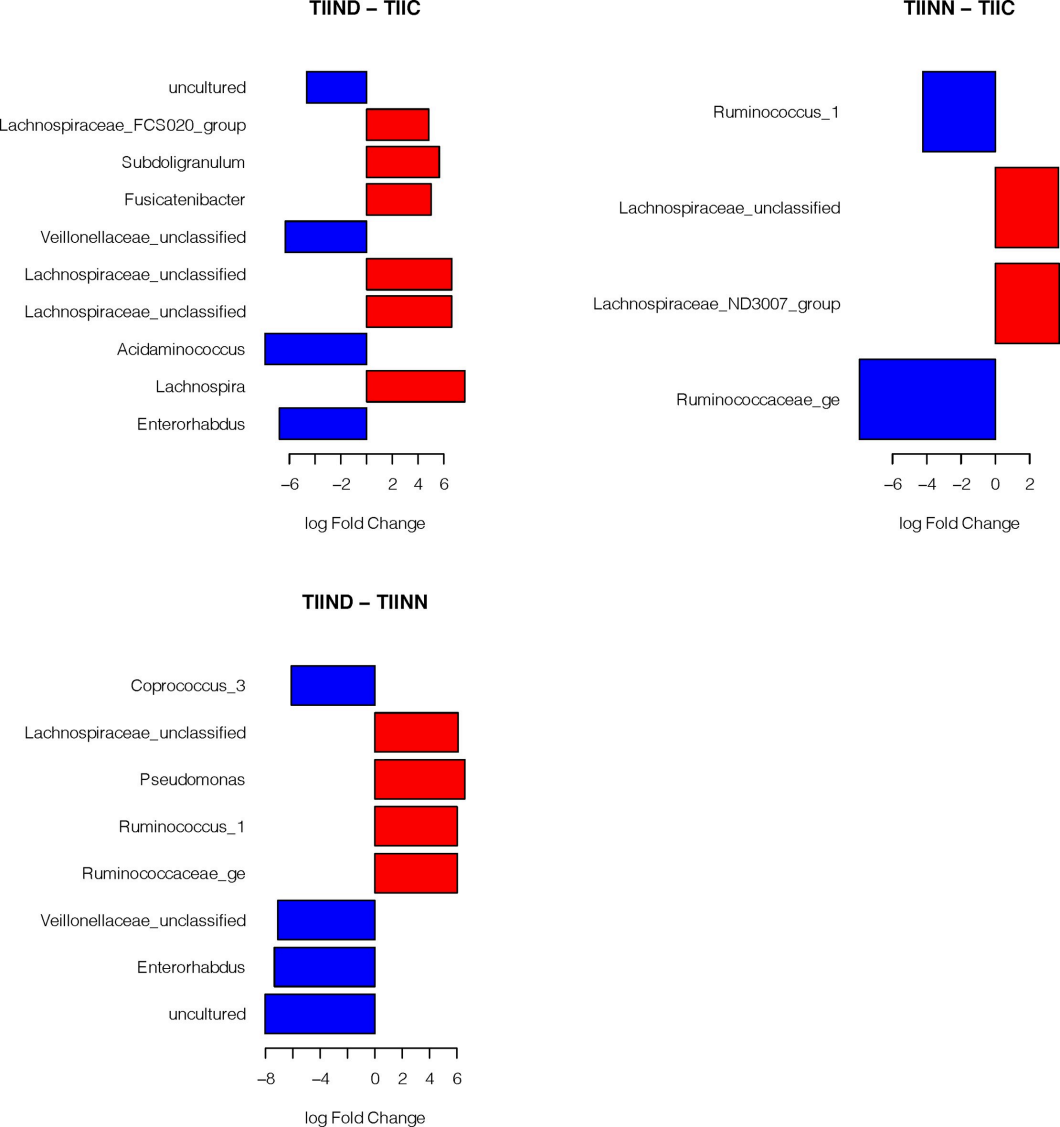
Log-fold-change between the different treatments for the type II diabetic subjects. Detailed results of the statistical testing are in S3 Table. * Abbreviations in Table 2.

The log-fold-change between the different treatments for the type II diabetic subjects is shown in Fig 5. It is interesting to show that there were differences in the trends in the log fold changes between the different treatments indicating that dysbiosis might be an important risk factor associated with TIID.

**Fig 6:**
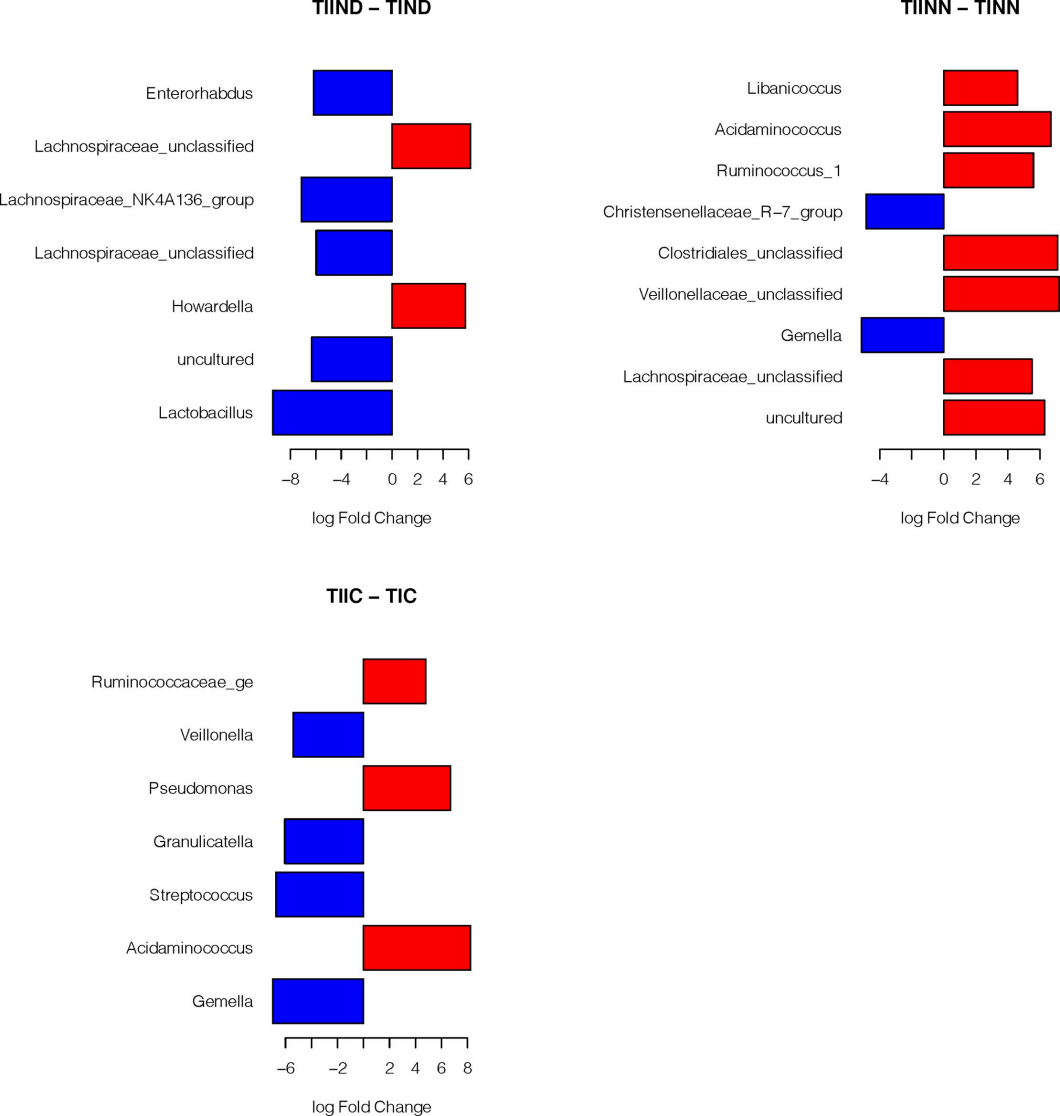
The log-fold-change of matched treatments between type I and type II diabetic subjects. Detailed results of the statistical testing are in S3 Table. * Abbreviations in Table 2.

Finally, Fig 6 compares the matched treatments between the type I and type II diabetic subjects. We note that TIIND shows higher abundance than TIND for Lachnospiraceae and *Howardella*, while for TIINN and TINN, Christensenellaceae and *Gemella* were more abundant in TINN. Both types of diabetes with controlled blood glucose level (TIC and TIIC) show different patterns of presence and abundance of bacterial families, mainly there is a shift towards the phylum *Firmicutes* (Ruminococcaceae and *Acidaminococcus*) in the TIIC treatment. We can observe that *Gemella* shows higher abundance in TINN and TIC than TIINN and TIIC.

## Discussion

Fecal samples were obtained from 47 subjects and used to evaluate the differences in microbiota composition in healthy controls and diabetic patients using 16S rRNA amplicon sequencing. The small sample sizes and the large variability observed between patients resulted in an inability to identify differences in Alpha and Beta diversity between the different treatment levels. However, even with such small sample sizes, we could identify OTUs that had differential relative abundance, indicated by significant log-fold changes between the compared treatments.Alldiabetic groups showed an increase in the abundance of Gram negative, potentially opportunistic pathogenic taxa (*Pseudomonas*, *Prevotella*) and Gram positive, *Gemella*. These bacteria might potentiate the pathogenesis of type II diabetes through production of endotoxins that induce inflammatory factors [33]. On the other hand, *Terrisporobacter* and *Turicibacter* were observed to be significantly more abundant in the control group as compared to either of the diabetes groups (Fig 3). These are highly fermenting bacteria [34,35] and future studies should be performed to understand their metabolic pathways and its effect on diabetes.

Based on our results we hypothesize that dysbiosis and disruption of the *Firmicutes*/*Bacteroidetes* ratio may have more impact on diabetes than the presence of a specific bacterial taxa. This dysbiosis is observed in many metabolic disorders such as obesity and diabetes [36,37]. Several studies have reported microbial signature in TIID. Tilg *et al*. [36] reported that intestinal dysbiosis in TIID was characterized by a decrease in *Roseburia intestinalis* and *F*. *prausnitzii* suggesting a direct link between an altered microbiota composition and the inflammatory state in patients with TIID. Also, a study done by Sircana *et al* [38] reported differences between the gut microbial composition in healthy individuals and those with TIID. These changes in the intestinal ecosystem could cause inflammation, alter intestinal permeability, and modulate metabolism of short-chain fatty acids, bile acids and metabolites that act synergistically on metabolic regulation systems contributing to insulin resistance. Interventions that restore equilibrium in the gut appear to have beneficial effects and improve glycemic control. In contrast to the previous studies, a study done on Egyptian citizens, by Salah *et al*. [37] concluded that obesity and diabetes were associated with enriched populations of both Firmicutes and Bacteroidetes. This could be explained by the type of diet, high carbohydrate intake and is correlated with high populations of Firmicutes and Bacteroidetes, while high fat diet is correlated withhigh abundance of Firmicutes only. Another study by Qin *et al*. [39] reported a moderate degree of dysbiosis in diabetic subjects compared to controls including reduction in the abundance of various *Firmicutes*; butyrate producing bacteria such as *Clostridiales*, *Eubacterium rectale*, *Faecalibacterium prausnitzii*, and *Roseburiain testinalis*. Additionally, they identified more opportunistic pathogens such *as*, *Clostridium hathewayi*, *Clostridium ramosum*, *Clostridium symbiosum*, *Bacteroides caccae*, *Escherichia coli and Eggerthella lenta*. These observations were consistent with our study, as the control group shows higher abundance of potentially beneficial bacterial genera (Fig 3) including *Lactobacillus*, *Terrisporobacter* and *Turicibacter*. These bacterial families are included in the *Firmicutes* phylum, which is known for its production of anti-inflammatory short chain fatty acids [40].

Recent studies suggest a link between type I diabetes and the gut microbiota [41,42]. In a study on rats, Brugman *et al*. [41] showed that, before onset of TID, composition of the gut microbiota was markedly different between rats that eventually developed TID and those that did not. Similarly, Roesch *et al*. [43] observed a significant decrease in abundance of *Lactobacillus*, *Bryantella*, *Bifidobacterium*, and *Turicibacter* taxa in Bio-Breeding Diabetes-Prone (BB-DP) rats, whereas abundance of *Bacteroides*, *Eubacterium*, and *Ruminococcus* increased in BB-DP rats compared to Bio-Breeding Diabetes-Resistant (BB-DR) rats. Thesefindings are consistent with our study where *Turicibacter*, *Terrisporobacter* and *Clostridium* were more abundant in the control group than in TID (Fig 3). In addition, in comparing different treatments of TID, it was observed that *Pseudomonas* (a Gram negative, opportunistic pathogenic taxon) was more prominent in the non-controlled blood glucose level (TINN) than in TIC (Fig 4).

Results from this pilot study should be taken cautiously as a basis for further investigations to understand differences in the microbiome in association with TID and TIID in Egyptian patients with controlled and non-controlled blood glucose and the presence or absence of liver disease onset or hyper-tension. In addition to the small sample sizes, there were other factors that we couldn’t control for in this study such as the type of diet. A high fat diet may shift the balance of the microbiota to taxa that cause inflammation, modulate metabolism and alter production of SCFA by microbiota leading to insulin resistance [44]. We aim to develop future studies, including larger samples sizes, to further elucidate our findings and alleviate some of these limitations, as well as possible metagenomic studies to examine the intriguing function of significant microbes.

## Supporting information

S1 Table Summary of factors associated with human subjects. (DOCX)

S2 Table This table provides the matched sample name and the disease condition and was used to facilitate analysis and comparison of each of these conditions. (CSV)

S3 Table This table shows compared treatments (contrasts), log fold changes between these compared treatments, p-values, FDR adjusted p-values along with the taxonomic assignment of the OTUs found significant at the 0.05 level. (CSV)

S1 Fig Rarefaction curves showing that the depth of sequencing per processed sample was adequate to capture the underlying diversity within these samples. (PDF)

S2 Fig Nonmetric multidimensional scaling (NMDS) with overlapping 95% confidence ellipsoids indicating no significant differences between the microbial community structure in the different treatment levels. Data points are marked with “*”. * Abbreviations in Table 2.
(PDF)

S3 Fig Bar plots representing the relative abundance of families observed with abundance more than 1% and aggregated over all samples per treatment level. (PDF)
