# Supplementary material for: A comparative study of the gut microbiome in Egyptian patients with Type I and Type II diabetes
Source: PLoS One. 2020 Sep 9;15(9):e0238764. doi: 10.1371/journal.pone.0238764 (PMC7480833; doi:10.1371/journal.pone.0238764)
Supplement: S1 Table — (DOCX) [file pone.0238764.s001.docx]

**S1 Table. Summary of factors associated with human subjects**

| **Item** | **TID**  **No. (%)** | **TIID**  **No. (%)** | **Healthy control**  **No. (%)** |
| --- | --- | --- | --- |
| **Male** | 8 (17%) | 12 (25.5%) | 2 (4%) |
| **Female** | 14 (30%) | 6 (13%) | 5 (10.5%) |
| **Age** | 33-62 | 32-62 | 24-57 |
| **Controlled blood glucose level- Diabetic only** | 8 (36.3%) | 6 (33.3%) |  |
| **Not controlled blood glucose level- Diabetic with liver diseases** | 4 (18.1%) | 2 (11.1%) |  |
| **Not controlled blood glucose level- Diabetic with hypertension** | 3 (13.6%) | 3 (16.6%) |  |
| **Not controlled blood glucose level- Diabetic only** | 7 (31.8%) | 7 (38.8%) |  |
| **Total** | 22 | 18 | 7 |

***TID; Type I diabetes, TIID; Type II diabetes***
