# Supplementary figures and images for: A comparative study of the gut microbiome in Egyptian patients with Type I and Type II diabetes
Source: PLoS One. 2020 Sep 9;15(9):e0238764. doi: 10.1371/journal.pone.0238764 (PMC7480833; doi:10.1371/journal.pone.0238764)

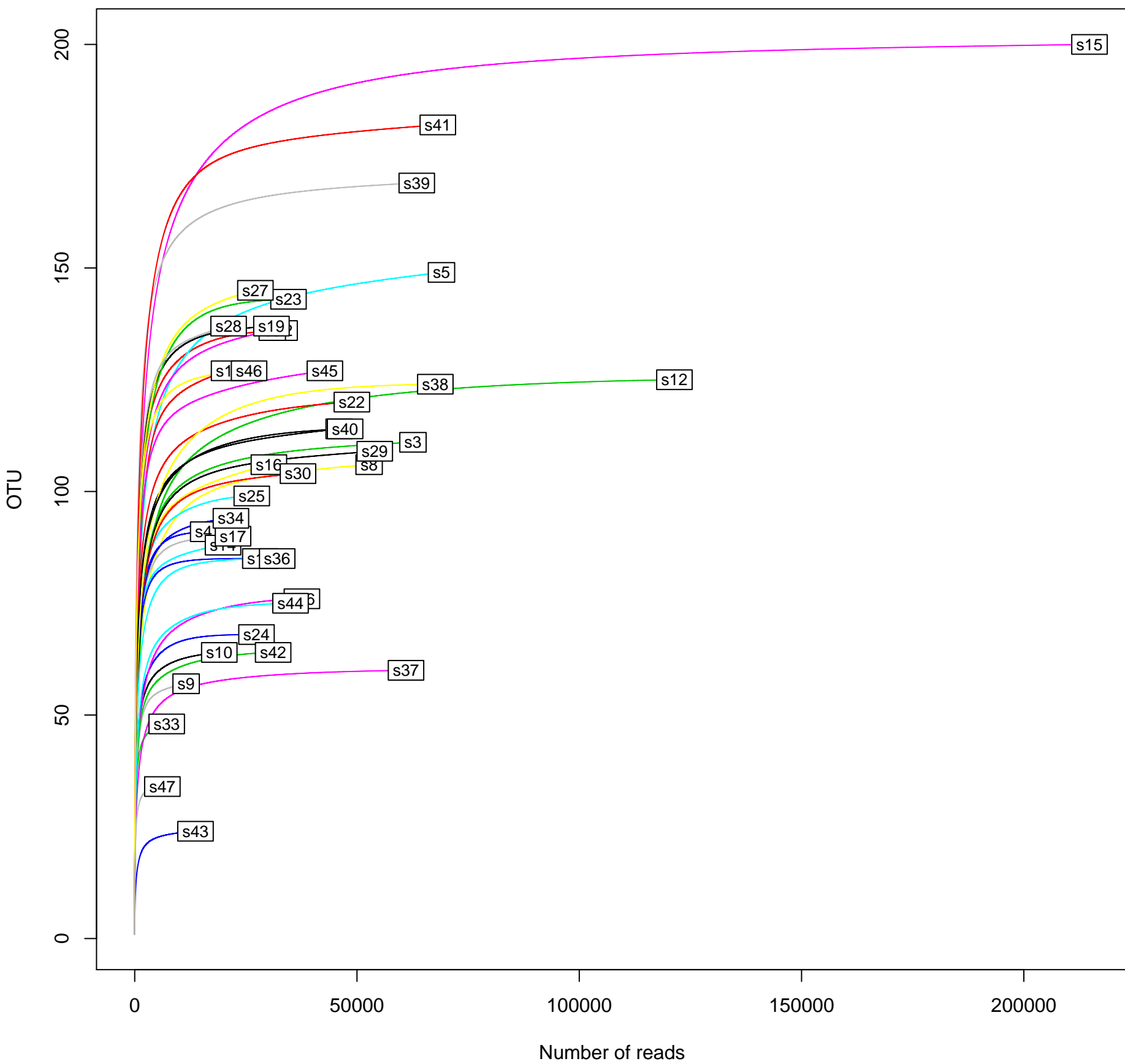

Supplement: S1 Fig — (PDF) [file pone.0238764.s004.pdf]

# Treatment

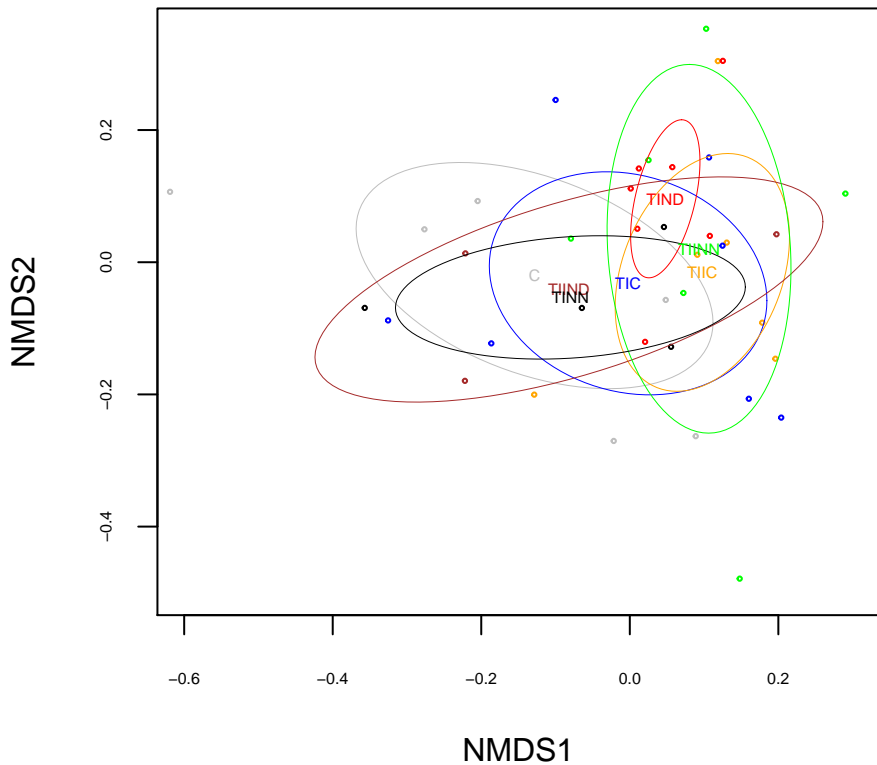

Supplement: S2 Fig — Data points are marked with “*”. * Abbreviations in Table 2. (PDF) [file pone.0238764.s005.pdf]

Barplot

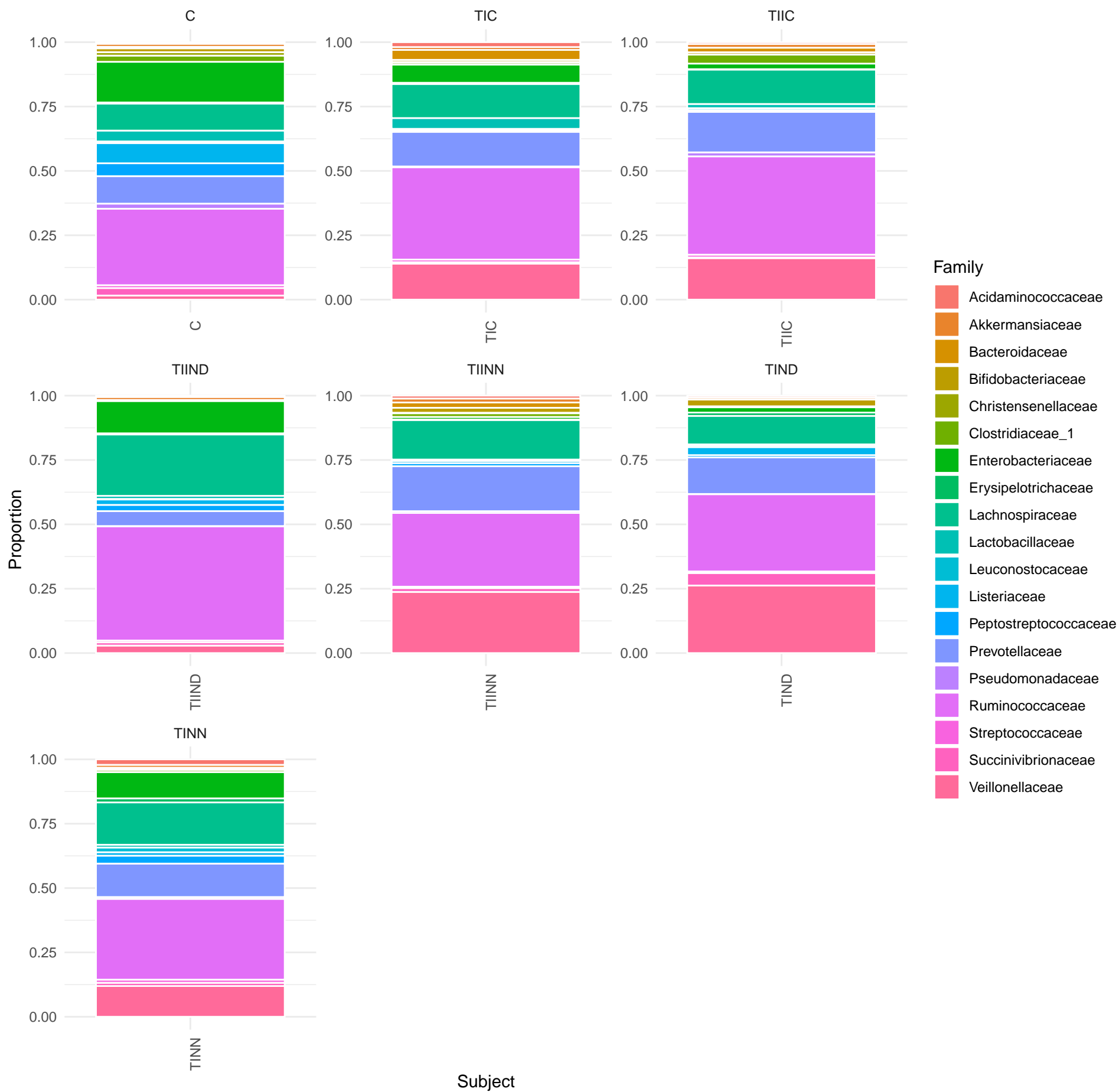

Supplement: S3 Fig — (PDF) [file pone.0238764.s006.pdf]
